# India’s DNA synthesis landscape: regulatory frameworks, industry structure, and biosecurity gaps

**DOI:** 10.3389/fbioe.2026.1879032

**Published:** 2026-10-19

**Authors:** Suryesh Kumar Namdeo, Mayra Ameneiros

**Affiliations:** 1 Indian Institute of Science, Bangaluru, Karnataka, India; 2 International Biosecurity and Biosafety Initiative for Science (IBBIS), Geneva, Switzerland

**Keywords:** biosecurity, customer screening, DNA synthesis, India, industry landscape, regulatory landscape, sequence screening, synthetic DNA

## Abstract

India is among the world’s largest and fastest-growing bioeconomies and ranks third globally in the number of in-country custom DNA synthesis providers, yet it lacks a dedicated regulatory framework governing the synthesis, sale, and distribution of synthetic nucleic acids. This perspective article examines India’s policy and regulatory landscape for synthetic DNA alongside a comprehensive mapping of the domestic DNA synthesis industry.

## 1 Introduction

As an increasingly significant commodity, synthetic nucleic acids constitute a foundation for applications ranging from basic research to disease detection. The synthesis of nucleic acids is becoming increasingly decentralized as costs, technology, and knowledge barriers decrease. As a result, over 700 companies worldwide are involved in selling, reselling, and distributing customized synthetic DNA (The International Biosecurity and Biosafety Initiative for Science; Grand View Research, 2026). The increasing consumption and expanding applications of synthetic DNA carry inherent biosecurity risks: the potential misuse of DNA synthesis to engineer or enhance harmful pathogens and biological agents (Williams and Kane, 2023).

Consistently enforced customer and sequence screening of synthetic DNA orders represents the most direct and effective mechanism for mitigating these risks, ensuring that access to potentially sensitive genetic material is restricted to verified users with demonstrably legitimate purposes (DiEuliis et al., 2017). Therefore, it is essential to establish strong global standards and policy frameworks to ensure DNA synthesis is safeguarded. However, the current regulatory landscape remains highly fragmented, with many laws and regulations outdated or insufficient (The World Economic Forum and Nuclear Threat Initiative, 2020; Asia Centre for Health Security), where screening practices are not consistently implemented worldwide.

For many years, despite widespread recognition among biosecurity experts of the potential risks associated with DNA synthesis, there has been no comprehensive global assessment identifying the companies involved in the production, sale, resale, and distribution of synthetic DNA. To address this gap, the Global DNA Synthesis Map (The International Biosecurity and Biosafety Initiative for Science), published by The International Biosecurity and Biosafety Initiative for Science (IBBIS) in 2025, was developed to provide the first publicly available global overview of the companies involved in DNA synthesis alongside the policy frameworks that govern them. Furthermore, significant gaps remain in understanding the extent to which these companies implement customer and sequence screening, the specific screening practices and procedures they employ, and the mechanisms in place for reporting suspicious orders to the relevant authorities (Kane and Parker, 2024).

While these gaps are now evident globally, understanding how they manifest in specific national contexts is equally important. This article therefore focuses specifically on India, examining the national policy landscape alongside research mapping DNA synthesis companies operating within the country. India provides a particularly relevant case study. It is one of the largest and fastest-growing bioeconomies in the world, expanding from approximately USD 10 billion in 2014 to over USD 165 billion in 2024, with projections reaching USD 300 billion by 2030 (Suresh et al., 2025).

The country’s extensive pharmaceutical and biotechnology sectors contribute significantly to both national economic growth and global supply chains. Often referred to as the “*pharmacy of the world*,” India plays a major role in the production of vaccines and generic medicines worldwide (Partsakoulaki, 2025). In addition, India has a vast and active life sciences ecosystem, with thousands of researchers and technicians working across academic institutions, national laboratories, hospitals, and industry in fields such as molecular biology, biochemistry, disease diagnostics, and agricultural biotechnology.

Synthetic DNA is a critical input for a wide range of foundational experiments, driving strong demand across India’s research and biotechnology sectors. India’s DNA synthesis industry is concentrated in key innovation hubs such as New Delhi, Bengaluru, and Hyderabad, reflecting the broader geographic clustering of the country’s biotechnology and life sciences sectors. These hubs bring together biotechnology companies, research institutions, universities, and other actors that contribute to the growing ecosystem for synthetic biology and related technologies. This perspective article presents the key findings of our research, providing an integrated assessment of India’s policy and regulatory landscape, the DNA synthesis industry, and existing approaches to customer and sequence screening.

Conducted as part of the IBBIS Global DNA Synthesis Map (The International Biosecurity and Biosafety Initiative for Science), the study draws on open-source research supplemented by targeted surveys used to validate and confirm the information collected. Together, these insights provide a high-level yet practical overview of the ecosystem, helping to inform strategies aimed at strengthening biosecurity measures in India.

## 2 Policy and regulatory landscape

India currently lacks a dedicated legal or regulatory framework specifically governing the synthesis, sale, distribution, and use of synthetic DNA. India currently has no specific regulatory requirement mandating the screening of synthetic DNA orders intended for domestic use. However, two existing sets of laws and regulations provide frameworks under which DNA synthesis activities and their potential misuse could be considered by relevant authorities.

The first one is the Environment Protection Law of 1986 and the associated 1989 regulation titled ‘Rules for the manufacture, use/import/export and storage of hazardous microorganisms/genetically engineered organisms or cells’ (Ministry of Environment and Forests, 1989). These rules created an initial institutional structure relevant to DNA synthesis with several competent authorities at different levels: 1) Recombinant DNA Advisory Committee, 2) Review Committee on Genetic Manipulation (RCGM), 3) Institutional Biosafety Committees (IBSCs), 4) Genetic Engineering Approval Committee, and 5) State and District level authorities. Under these regulations, the production of genetically engineered products is prohibited without the approval of the Genetic Engineering Approval Committee.

These rules established a list of pathogens classified into different risk groups, which has been revised and updated several times over the years. These rules and the follow-up guidelines on the IBSCs in 2011 and 2020 have empowered the IBSCs to make decisions on several matters, including safety and security issues related to DNA, according to the surveys conducted in this research (Department of Biotechnology Government of India and Biotech Consortium India Limited, 2011; Department of Biotechnology Government of India, 2020a). Further, the revised and simplified ‘Guidelines on Import, Export and Exchange of GE organisms and products thereof for R&D purposes’ set conditions for the IBSC and the RCGM, and approvals for trade and exchange of nucleic acids which include synthetic DNA (Department of Biotechnology, Government of India, 2020b).

In 1990, another set of rules and guidelines was implemented to establish detailed biosafety regulations in India, and was subsequently revised in 2017. These were called “Regulations and Guidelines on Biosafety of Recombinant DNA Research and Biocontainment”; they provide guidance on biocontainment and quality control to ensure the safety of recombinant DNA products (Department of Biotechnology Government of India; Department of Biotechnology Government of India, 2017). These guidelines lack any specific mention of DNA synthesis but provide an updated list of infectious organisms corresponding to different risk groups whose DNA could be screened. None of these rules or regulations include specific provisions or requirements related to customer screening or DNA sequence screening.

It is important to note that the IBSC clearance requires researchers to provide comprehensive information about all genetic experiments they are conducting, including the use of synthetic DNA. The IBSC clearance is required for all entities conducting genetic experiments, including academic institutes, national laboratories, and industry.

Under India’s Special Chemicals, Organisms, Materials, Equipment and Technologies (SCOMET) export control list, genetic elements that could enhance the pathogenicity of listed microorganisms are implicitly regulated, particularly where they pose a risk to human, animal, or plant health. The primary government agency in India responsible for developing and implementing these regulations is the Department of Biotechnology (DBT), which receives contextual inputs from the Indian Council of Medical Research (ICMR) and the Ministry of Environment, Forests, and Climate Change (MoEFCC).

Throughout the country, there have been several national biotechnology strategies, but they lack a special emphasis on addressing biosecurity risks. However, these risks do find rare mention in some state-level policies, such as the Karnataka State Biotechnology Policy 2024–2029 (Government of Karnataka Department of Electronics, Information Technology and Biotechnology, 2025). At the national level, DBT hosts the RCGM Secretariat, which is responsible for implementing several of these regulations. In recent years, DBT created the Indian Biosafety Knowledge Portal (IBKP), an interactive web portal that contains information on transactions dealing with the RCGM and the relevant rules and regulations (RCGM Secretariat).

This portal also facilitates the online submission of Forms for Import/Export/Research and development projects, pre-clinical toxicity of recombinant DNA-derived biosimilars, and Biosafety Research Level trials. All the IBSC clearances must be uploaded on IBKP to register and obtain an RCGM certificate.

The second set of laws under which misuse of synthetic DNA could be considered includes the Weapons of Mass Destruction (WMDs) and Their Delivery Systems (Prohibition of Unlawful Activities) Act of 2005 (amended in 2022) (Ministry of External Affairs, Government of India). This Act implements the provisions of the Biological Weapons Convention (BWC) in India and prohibits any material that could lead to the development or delivery of biological weapons. These provisions could potentially apply to synthetic DNA, although synthetic DNA is not explicitly referenced in the Act. It also prohibits any support to non-state actors for the development of WMDs, including bioweapons. However, currently there are no specific rules, regulations, or guidelines under this Act for biosecurity, which limits its effectiveness beyond deterrence.

Additionally, based on the misuse context, the Unlawful Activities (Prevention) Act, 1967 (amended in 2019) could be applied to prevent terrorist activities involving biological weapons (Ministry of Home Affairs). However, both laws focus on deterrence rather than providing specific measures to prevent the development of biological weapons, which remains a substantial regulatory gap.

In summary, India’s regulatory landscape for synthetic DNA remains fragmented and lacks a dedicated oversight framework. The country’s biosecurity architecture for nucleic acid synthesis instead rests on the collection of instruments previously mentioned, not originally designed with synthetic DNA in mind. The most concrete biosecurity instrument currently in place is the SCOMET export control list, which implicitly regulates genetic elements capable of enhancing the pathogenicity of listed microorganisms. Export licenses for controlled materials are required and issued by the Directorate General of Foreign Trade (DGFT), making the export control regime the primary—and enforceable—point of oversight. However, this mechanism applies exclusively at the border, leaving the domestic synthesis transaction itself entirely unregulated, with no obligation on synthesis providers to screen DNA sequences or customers, and for end users to order synthetic DNA from a provider that screens.

## 3 Industry landscape

India’s position as a major hub for DNA synthesis is evident from a global comparison when we analyze the total number of in-country custom nucleic acid companies. The United States leads with 111 companies offering custom synthesis services, followed by China with 77. India ranks third, with 31 in-country custom nucleic acid companies (The International Biosecurity and Biosafety Initiative for Science). This places India among the leading countries in the sector and highlights both the scale of its domestic activity and the urgency of addressing existing regulatory gaps, particularly in the absence of a dedicated oversight framework for synthesis practices.

Overall, India accounts for approximately 9% of the global share of custom nucleic acid companies, ranking third worldwide, based on the total number of such companies identified across 74 countries, and first within Central and Southern Asia. In total, 59 companies associated with nucleic acid services were identified in India. Of these 59 companies, only 31 conduct custom in-country DNA synthesis, while 21 operate as third-party vendors. The remaining companies provide nucleic acid-related services (such as sequencing) without performing synthesis. No manufacturers of benchtop synthesis devices were identified.

This distribution indicates a relatively strong domestic industry, characterized primarily by in-country synthesis providers. Many of the third-party vendors function as intermediaries, engaging in the resale of products—often sourced from large, predominantly US-based synthesis firms.

When it comes to screening practices, only a few companies have oligo order forms on their websites, which could be used to collect customer information with the possibility of conducting customer screening. General responses from surveys conducted in this study reveal a lack of sequence screening in Indian companies. Generally, only the name of the gene is required. Indian companies are not members of the International Gene Synthesis Consortium (IGSC) and do not follow its protocols at the time of writing this article.

Several IGSC member companies have Indian distributors and subsidiaries. However, whether they conduct customer and sequence screening in India could not be ascertained.

The combination of India’s status as a Major Synthesis Hub and its limited policy oversight represents a significant biosecurity concern flagged explicitly by the Global Synthesis Map (The International Biosecurity and Biosafety Initiative for Science) research, which identifies India as a priority country for policy advocacy. India has limited policies providing oversight of DNA synthesis: screening is implicit rather than mandatory, no formal screening framework exists, and while a control list and export license requirements are in place, these instruments do not impose proactive screening obligations on domestic synthesis providers. The gap between India’s substantial and growing industry footprint—first in its region, third globally—and its underdeveloped regulatory architecture makes it one of the most consequential oversight gaps in the global synthetic DNA landscape.

## 4 Policy recommendations and way forward

Overall, the policy landscape in India is fragmented and lacks specific provisions to address the biosecurity risks associated with synthetic DNA. Existing institutional mechanisms, particularly IBSCs and the RCGM framework, have historically focused primarily on biosafety concerns, such as preventing accidental exposure, laboratory releases, and environmental contamination. Although IBSCs have increasingly been assigned responsibilities that extend into biosecurity, this raises an important question regarding whether committee members have the specific expertise and training required to identify and assess risks of deliberate misuse. Evaluating intentional misuse requires a different set of considerations from conventional laboratory biosafety assessments. Consequently, relying on IBSCs to address these emerging biosecurity risks may be insufficient, particularly given the uneven implementation of IBSC mechanisms across institutions and the absence of dedicated requirements for synthetic DNA screening. There are guidelines on the trade and exchange of biological products, including nucleotides. However, in the absence of a national biosecurity strategy, a specific DNA synthesis screening framework, and a defined “sequences of concern” list, customer and sequence screening for biosecurity risks is not mandatory for companies based in India selling at a national level.

This poses a challenge when it comes to bringing different stakeholders together to discuss the biosecurity risks associated with synthetic DNA. It also creates uncertainty around roles, responsibilities, and best practices for risk mitigation. At the same time, it is important to avoid unnecessary overregulation that could hinder innovation and slow progress in the country. Striking the right balance will require clear guidelines, stakeholder coordination, and capacity-building efforts that support both responsible research and technological advancement. Further, regulations aimed at addressing these risks should adopt a multidisciplinary approach to effectively categorize risk levels and enable the development of tailored mitigation strategies.

The industry landscape analysis reveals that a significant number of companies conduct in-house gene and oligo synthesis in India, and several more are part of the distribution networks of largely U.S.-based companies. Orders fulfilled by U.S.-based IGSC members may already be screened before being distributed through Indian companies. Incentivizing Indian companies to screen would be important, but challenging, requiring a combination of policy and regulatory tools and the promotion of international norms and standards through market forces. Any substantial effort would require close engagement with industry partners such as the Association of Biotechnology Led Enterprises (ABLE), the domestic biotech industry body, to balance the management of security concerns with concerns regarding overregulation and financial costs.

Promotion of free, open-source, and globally available tools such as IBBIS’s Common Mechanism could play a crucial role in expanding access to DNA synthesis screening capabilities. At present, DNA synthesis screening represents one of the most practical and effective safeguards available to reduce the potential misuse of synthetic DNA. However, such tools should not be regarded as complete or static solutions. Rapid advances in AI-enabled protein design are creating new challenges for screening approaches that rely substantially on sequence similarity. For example, recent work by Microsoft researchers and collaborators, including IBBIS, demonstrated that generative protein design tools can produce substantially modified protein sequences that may evade conventional sequence-based screening approaches, prompting subsequent improvements to existing screening tools. This work also highlighted the broader limitation of using sequence similarity as a proxy for biological function and the potential need for increasingly function-aware screening approaches. This work led to the development and deployment of patches that substantially improved the detection of AI-generated variants designed to evade conventional screening. The Common Mechanism has similarly continued to evolve and is designed to provide greater resilience against screening-evasion attempts, including AI-generated sequences. Nevertheless, existing screening approaches may not detect all AI-generated sequences, highlighting the need for continued evaluation, red-teaming, and regular updates to screening tools as AI-enabled biological design capabilities continue to advance.

As concrete near-term steps, DBT should assume a central coordinating role, in consultation with DGFT and ICMR, and initiate the development of a national “sequences of concern” framework aligned with existing international frameworks such as the IGSC Harmonized Screening Protocol, along with a reporting mechanism for flagged orders. The IBSC mechanism should be strengthened by raising awareness of the potential misuse of synthetic DNA, building capacity on preventive measures, and establishing interagency coordination pathways. DBT should also develop biosecurity-specific guidance and training for IBSC members, with particular attention to distinguishing deliberate misuse risks from conventional biosafety concerns and establishing clear referral pathways for cases requiring expertise beyond the institutional level.

Further, the Bureau of Indian Standards (BIS) and DBT should establish a certification scheme for domestic synthesis providers that adopt sequence and customer screening. Implementation could initially be incentive-based, with public research funding and government procurement progressively favoring synthesis providers that demonstrate adherence to recognized customer and sequence screening practices. These steps would enable India to demonstrate biosecurity leadership commensurate with its global industry standing while avoiding the immediate costs of prescriptive legislative reform.

Importantly, implementation should be accompanied by measurable indicators rather than relying solely on voluntary commitments. These could include the proportion of domestic synthesis providers conducting both sequence and customer screening, participation in a national certification scheme, the number of relevant IBSCs receiving dedicated biosecurity training, and the establishment and use of mechanisms for reporting and escalating potentially concerning orders. DBT, together with the relevant agencies and industry stakeholders, should periodically assess progress against these indicators.

If voluntary adoption and market incentives fail to achieve sufficient screening coverage, India should consider progressively stronger measures, including linking compliance to public research funding, procurement, certification or licensing requirements, and, where necessary, introducing minimum mandatory customer and sequence screening requirements. Failure to address these gaps could otherwise increase vulnerabilities within the research and biotechnology ecosystem as domestic synthesis capabilities and emerging technologies continue to advance.

An incentive-based national approach could draw lessons from the United States. Rather than imposing a universal regulatory requirement on all providers and customers, the U.S. framework leverages federal funding and procurement mechanisms to promote compliance. Researchers receiving U.S. federal funding are expected to procure synthetic nucleic acids from providers that adhere to the federal screening framework, thereby creating a market-based incentive for synthesis providers to implement both sequence and customer screening. A comparable mechanism could provide India with an intermediate step between purely voluntary adoption and comprehensive mandatory regulation, while generating evidence on implementation, costs, and screening uptake.

The 2024 U.S. Framework for Nucleic Acid Synthesis Screening established expectations for sequence-of-concern screening, customer legitimacy assessment, reporting of potentially illegitimate orders, record retention, and procurement from providers adhering to the framework. However, the U.S. policy landscape itself remains under development. Executive Order 14,292, issued in May 2025, directed the Office of Science and Technology Policy (OSTP) to revise or replace both the 2024 Framework for Nucleic Acid Synthesis Screening and the 2024 U.S. Government Policy for Oversight of Dual Use Research of Concern and Pathogens with Enhanced Pandemic Potential. The Order further calls for comprehensive, scalable, and verifiable nucleic acid synthesis screening and requires the screening framework to be periodically reviewed and revised. The U.S. experience therefore provides a useful reference point for India while also demonstrating that national biosecurity frameworks must remain adaptable as technologies, risks, and screening capabilities evolve.

India’s current position may therefore be better understood as part of a broader global implementation gap in which synthesis-specific biosecurity considerations have yet to be fully incorporated into national governance frameworks. This interpretation is consistent with the approach proposed in “Compounding asymmetries in nucleic acid synthesis screening: policy pathways for strengthening global biosecurity governance, (Zakaria et al., 2026)” which argues that the adoption of nucleic acid synthesis screening should be understood as a continuum rather than as a binary distinction between countries that screen and those that do not. From this perspective, countries can progressively strengthen their screening capacity through awareness raising, voluntary screening practices, customer verification, institutional capacity building, incentives, and, where appropriate, eventual incorporation of screening requirements into national policy frameworks. This gradual approach is particularly relevant to India, where existing biotechnology governance structures provide potential entry points for strengthening synthetic DNA biosecurity without requiring immediate comprehensive legislative reform. However, progression along this continuum should be linked to defined institutional responsibilities, implementation milestones, measurable outcomes, and periodic review. For India, this could provide a staged pathway from capacity building and voluntary adoption, through certification and funding or procurement incentives, to minimum mandatory screening requirements if earlier measures prove insufficient.

## Data Availability

The datasets presented in this article are not readily available because of information hazards. Requests to access the datasets should be directed to www.globalsynthesismap.bio.
